# Optimising predictive modelling of Ross River virus using meteorological variables

**DOI:** 10.1371/journal.pntd.0009252

**Published:** 2021-03-09

**Authors:** Iain S. Koolhof, Simon M. Firestone, Silvana Bettiol, Michael Charleston, Katherine B. Gibney, Peter J. Neville, Andrew Jardine, Scott Carver

**Affiliations:** 1 College of Health and Medicine, School of Medicine, University of Tasmania, Hobart, Tasmania, Australia; 2 School of Natural Sciences, University of Tasmania, Hobart, Tasmania, Australia; 3 Melbourne Veterinary School, Faculty of Veterinary and Agricultural Sciences, University of Melbourne, Melbourne, Victoria, Australia; 4 Victorian Department of Health and Human Services, Communicable Disease Epidemiology and Surveillance, Health Protection Branch, Melbourne, Victoria, Australia; 5 Department of Health, Western Australia, Environmental Health Directorate, Public and Aboriginal Health Division, Perth, Western Australia, Australia; University of Queensland, AUSTRALIA

## Abstract

**Background:**

Statistical models are regularly used in the forecasting and surveillance of infectious diseases to guide public health. Variable selection assists in determining factors associated with disease transmission, however, often overlooked in this process is the evaluation and suitability of the statistical model used in forecasting disease transmission and outbreaks. Here we aim to evaluate several modelling methods to optimise predictive modelling of Ross River virus (RRV) disease notifications and outbreaks in epidemiological important regions of Victoria and Western Australia.

**Methodology/Principal findings:**

We developed several statistical methods using meteorological and RRV surveillance data from July 2000 until June 2018 in Victoria and from July 1991 until June 2018 in Western Australia. Models were developed for 11 Local Government Areas (LGAs) in Victoria and seven LGAs in Western Australia. We found generalised additive models and generalised boosted regression models, and generalised additive models and negative binomial models to be the best fit models when predicting RRV outbreaks and notifications, respectively. No association was found with a model’s ability to predict RRV notifications in LGAs with greater RRV activity, or for outbreak predictions to have a higher accuracy in LGAs with greater RRV notifications. Moreover, we assessed the use of factor analysis to generate independent variables used in predictive modelling. In the majority of LGAs, this method did not result in better model predictive performance.

**Conclusions/Significance:**

We demonstrate that models which are developed and used for predicting disease notifications may not be suitable for predicting disease outbreaks, or *vice versa*. Furthermore, poor predictive performance in modelling disease transmissions may be the result of inappropriate model selection methods. Our findings provide approaches and methods to facilitate the selection of the best fit statistical model for predicting mosquito-borne disease notifications and outbreaks used for disease surveillance.

## Author summary

Mosquito-borne diseases cause significant illness worldwide. Mosquito breeding, which leads to disease transmission, is driven by favorable climatic and meteorological events (e.g., rainfall and warm temperatures). Understanding the association meteorological conditions have with mosquito breeding aids in directing mosquito control activities when there is a likelihood of disease transmission. Predictive models are used in public health decision making and resource allocation to guide mosquito control programs. However, there are multiple modelling methods, all of which provide differing degrees of accuracy in their predictions and suitability to the disease transmission dynamics. This study aims to assess commonly used statistical models for predicting mosquito-borne disease notifications and outbreaks. We demonstrate that statistical model selection plays an important role in accurately forecasting mosquito-borne disease and poor predictive performance may be due to inappropriate model selection. Furthermore, a model suited to predicting disease notifications may not always be the best model to accurately predict the occurrence of disease outbreaks. The methods used here can aid in public health to establish suitable predictive mosquito-borne disease surveillance systems to help guide disease prevention and resource allocation, and mosquito control activities.

## Introduction

Meteorological factors influence the transmission ecology of pathogen, host and vector species populations, and human behaviour, which can act directly or indirectly to drive mosquito-borne disease dynamics [1,2]. Climate events (such as rainfall or tidal events) impact upon mosquito population dynamics and the presentation of disease in host and human populations preceding these events. The time between meteorological events that lead to increases in mosquito populations and when mosquito-borne diseases are detected in humans represents the enzootic transmission cycle. This period includes the diseases’ intrinsic incubation period and the circulation through animal populations before transmission spilling over into human populations. The time delay preceding meteorological events (e.g., heavy rainfall), which represents the circulation and transmission of disease before the spillover into humans, make mosquito-borne diseases well suited for predictive modelling (i.e., forecasting) of outbreaks. There are several statistical methods that are suited for forecasting disease notifications [1,3–6]. Differing predictive modelling approaches in the literature likely vary in their ability to predict disease activity, but it is unknown which methods are better and under which circumstances. In this study, we address this problem by assessing commonly used statistical methods in forecasting mosquito-borne disease notifications and outbreaks in Australia.

Ross River virus (RRV, family *Togaviridae*, genus *Alphavirus*) is an important arbovirus that is endemic in Australia having a complex epidemiology with a multi-vector and multi-host transmission system being dependent on ecological context [7–10]. It is the most common mosquito-borne virus affecting humans in Australia, with an annual average incidence rate of 40 cases per 100,000 population [11]. Over the past two decades, epidemiological studies on environmental and meteorological factors have been conducted across multiple regions of Australia, providing insight into the factors and complexity of RRV transmission across different locations [1,2,6,12–15]. The variations reported include site-specific meteorological, environmental, and geographic factors, mosquito vector species, and host species [7–9].

There are multiple time series statistical modelling studies aimed at forecasting RRV transmission. Epidemiological analyses have typically focused on locations where attack rates of RRV are highest and areas where transmission is seasonally driven with either an annual or bi-annual oscillation of human disease cases [6,15]. Statistical models predicting RRV notifications include, but are not limited to: logistic and Poisson regressions, negative binomial regressions, seasonal and non-seasonal auto-regressive integrated moving average models, and generalised additive models [e.g.,1,2,6,13–18]. The use of these models has primarily been to estimate the probability of an RRV outbreak at a given time, or to predict counts of notifications using a combination of environmental and meteorological factors, and mosquito surveillance [e.g.,13,17]. The sensitivity and specificity of predicting outbreaks in previous forecasting studies vary, yet there has been, to our knowledge, no evaluation of the relative performance of the types of models used in forecasting RRV. Of studies that have focused on predicting RRV transmission, few present the models’ predictive performance [10].

The aim of this paper is to evaluate several modelling methods for predicting RRV notifications and outbreaks using meteorological variables, and to assess factors affecting predictive performance. These include generalised boosted regression, generalised additive regression, hurdle regression, negative binomial regression, and auto-regressive integrated moving average regression models. To maximise the utility of the study, we undertook the forecasting across sites in Victoria and Western Australia that include locations with a varying number of RRV notifications and are subject to systematic meteorological and vector population monitoring. At each site, we model both RRV notifications per 100,000 population and the likelihood of a disease outbreak as these are desired forecasting outputs to inform public health policy in Australia. We follow a systematic approach to develop a framework in constructing and selecting the best performing epidemiological models.

## Methods

### Data

**Fig 1:**
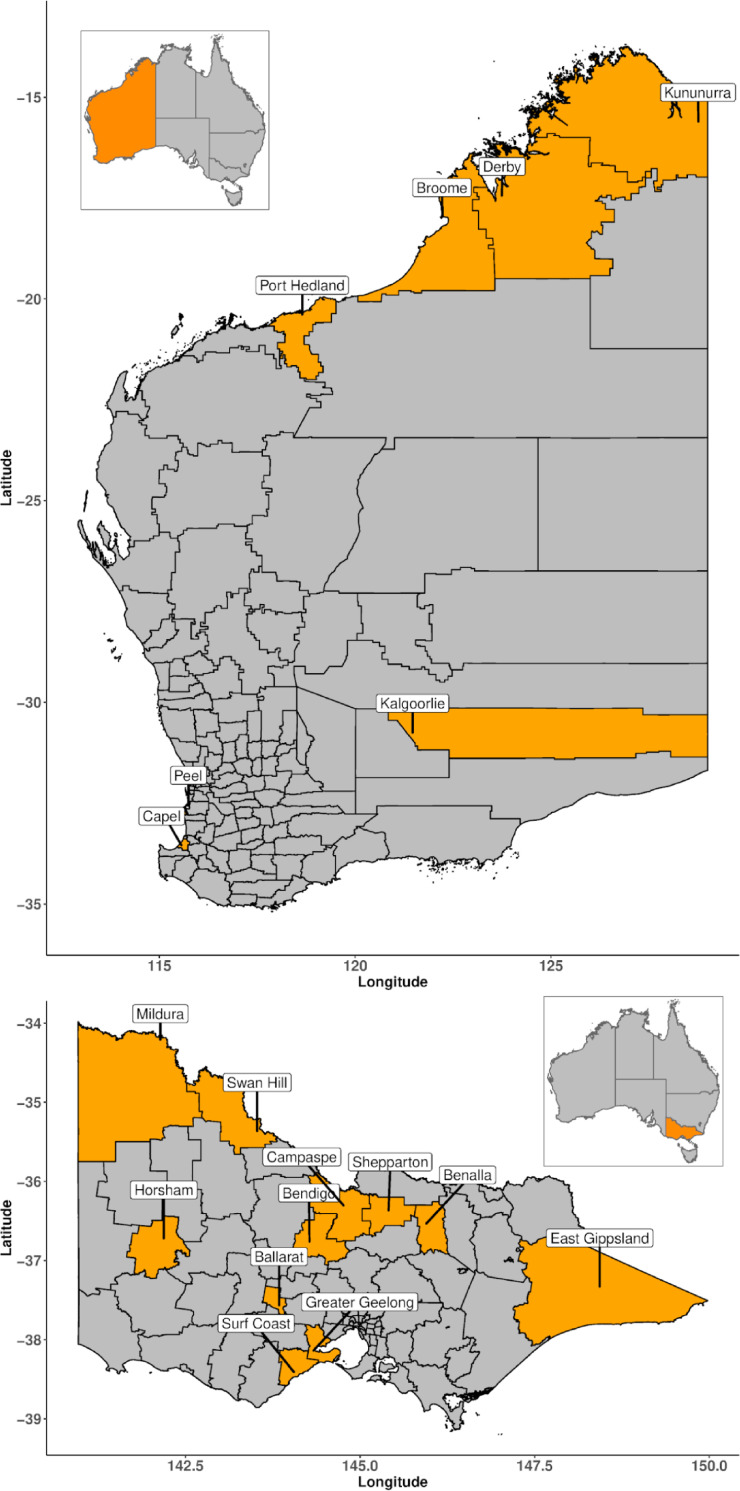
Local Government Areas used in forecasting Ross River virus notifications and outbreaks across the States of Victoria and Western Australia, Australia.

This study included 18 sites that experience RRV outbreaks; sites included 11 Victorian and seven Western Australian Local Government Areas (LGAs) (Fig 1). RRV notifications for Victoria and Western Australia were extracted from the Public Health Event Surveillance System (PHESS) held within the Victorian Department of Health and Human Services, and the Western Australian Notifiable Infectious Diseases Database (WANIDD) held by Western Australian Department of Health, respectively. RRV notification data included the estimated month or week and year of RRV symptom onset, postcode and, for Victoria only, serological testing results for the RRV infection. RRV notifications were aggregated into the total number of notifications by month and year. Notifications of RRV were included if they met the most recent national surveillance case definition for confirmed or probable RRV (effective 1st January 2016): specifically, detection of RRV by polymerase chain reaction (PCR) or demonstration of RRV-IgG seroconversion for confirmed RRV, or detection of both RRV-IgM and RRV-IgG within the same specimen for probable RRV [19]. Ross River virus human notification data were collected from July 2000 until June 2018 in Victoria, and from July 1991 until June 2018 in Western Australia. Population estimates for each LGA were obtained from the Australia Bureau of Statistics [20].

Meteorological data were collected from the SILO database hosted by the Queensland Government, which provides access to daily meteorological datasets of a range of meteorological and climate variables from Bureau of Meteorology weather stations [21]. All variables examined were summarised into monthly observations for each LGA (Table 1) which included (per month): total rainfall (mm), maximum and minimum temperature (degrees Celsius), mean vapor pressure (hPa), maximum and minimum relative humidity (%), Morton’s areal actual and potential evapotranspiration (mm), and mean sea level pressure (hPa). These variables were chosen based on their availability and use in previous RRV forecasting studies. Where multiple weather stations existed within a single LGA, we used a single weather station closest to the main population centre where the majority of RRV notifications were reported. The use of a single station was also necessary due to a high rate of intermittent and discontinuous monitoring by the other stations outside of population centres.

**Table 1:** Best fit model predictive performance of RRV notifications and outbreaks in local government areas (LGA) in Victoria (VIC), and Western Australia (WA) by LGA climate. The total number of RRV notifications (Cases), the best model used for predicting RRV notifications, adjusted R-squared coefficient (R^2^), the best model used for predicting outbreaks, sensitivity (Sn), specificity (Sp), and Matthews correlation coefficient (MCC). ARIMA = auto-regressive integrated moving average model; GAM = generalised additive model; BR = generalised boosted regression; NB = negative binomial regression; and Hurdle = hurdle regression. Ninety five percent confidence intervals (95% CI) are given of the distribution of each predictive performance measure from Jackknife pseudo-random sampling using the respective best fit model. Models with a “*” following the model type used the Factorial Approach. See Table 2 for a comparison of how close modelling methods were to one another for predicting RRV notifications and outbreaks.

				Notification Models		Outbreak Models			
State	LGA	Climate	Cases (n)	Best model	R^2^ (95% CI)	Best model	Sn (95% CI)	Sp (95% CI)	MCC (95% CI)
VIC	Ballarat	Temperate	65	GAM	0.533 (0.505–0.534)	GAM	0.75 (0.67–0.69)	1.00 (0.98–0.99)	0.86 (0.75–0.76)
VIC	Benalla	Semi-arid	65	GAM	0.141 (0.109–0.147)	GAM*	0.50 (0.48–0.53)	0.89 (0.89–0.89)	0.26 (0.22–0.25)
VIC	Bendigo	Semi-arid	170	BR	0.431 (0.154–0.247)	BR	0.25 (0.29–0.31)	1.00 (1.00–1.00)	0.49 (0.49–0.50)
VIC	Campaspe	Semi-arid	205	Hurdle	0.757 (0.725–0.745)	Hurdle	0.50 (0.50–0.51)	1.00 (1.00–1.00)	0.70 (0.70–0.70)
VIC	Geelong	Semi-arid	103	Hurdle	0.581 (0.340–0.508)	Hurdle	0.25 (0.21–0.23)	1.00 (1.00–1.00)	0.49 (0.37–0.41)
VIC	Gippsland	Semi-arid	126	GAM*	0.214 (0.195–0.219)	Hurdle	0.43 (0.37–0.41)	0.92 (0.92–0.92)	0.31 (0.29–0.30)
VIC	Horsham	Semi-arid	205	BR	0.143 (0.060–0.143)	BR	0.67 (0.63–0.64)	0.96 (0.96–0.96)	0.49 (0.49–0.50)
VIC	Mildura	Temperate	312	Hurdle	0.462 (0.303–0.418)	GAM	0.25 (0.30–0.32)	1.00 (0.99–1.00)	0.49 (0.27–0.28)
VIC	Shepparton	Temperate	201	GAM	0.301 (0.203–0.391)	GAM	0.25 (0.16–0.19)	1.00 (1.00–1.00)	0.49 (0.29–0.33)
VIC	Surf Coast	Temperate	98	GAM	0.078 (0.037–0.120)	Hurdle	0.25 (0.22–0.24)	0.99 (0.96–0.97)	0.33 (0.21–0.23)
VIC	Swan Hill	Temperate	128	GAM	0.065 (0.055–0.070)	GAM	0.00 (0.00–0.00)	1.00 (1.00–1.00)	0.00 (0.00–0.00)
WA	Broome	Semi-arid	542	BR	0.518 (0.325–0.449)	BR	0.75 (0.67–0.71)	0.95 (0.95–0.95)	0.54 (0.48–0.50)
WA	Capel	Temperate	305	NB	0.226 (0.210–0.233)	NB	0.00 (0.00–0.01)	0.96 (0.96–0.97)	0.00 (-0.02 - -0.01)
WA	Derby	Semi-arid	100	GAM	0.357 (0.334–0.386)	NB	0.43 (0.36–0.37)	0.99 (0.97–0.98)	0.54 (0.43–0.44)
WA	Kalgoorlie	Temperate	264	NB	0.145 (0.130–0.156)	NB	0.00 (0.00–0.00)	1.00 (1.00–1.00)	0.00 (0.00–0.00)
WA	Kununurra	Semi-arid	178	BR	0.382 (0.355–0.397)	BR	0.80 (0.77–0.78)	0.90 (0.90–0.91)	0.50 (0.49–0.50)
WA	Peel	Temperate	2044	Hurdle*	0.245 (0.150–0.324)	BR*	0.18 (0.17–0.18)	0.97 (0.95–0.96)	0.24 (0.18–0.19)
WA	Port Hedland	Semi-arid	196	Hurdle	0.176 (0.132–0.151)	GAM	1.00 (0.61–0.69)	0.88 (0.89–0.90)	0.40 (0.23–0.27)
Mean									
Overall					0.320		0.40	0.97	0.43
VIC					0.337		0.37	0.98	0.50
WA					0.293		0.45	0.95	0.32
STDEV									
Overall					0.195		0.29	0.04	0.17
VIC					0.231		0.26	0.03	0.21
WA					0.132		0.30	0.04	0.10

## Statistical analysis

Statistical analysis followed a stratified structured approach when linking meteorological predictors with RRV notifications. We undertook two approaches in constructing predictive models; the first used meteorological factors as independent variables within the predictive models, hereafter referred to as the “Independent Approach”. The second approach used factor analysis to determine factor scores of the meteorological variables to be used as independent variables in the models, hereafter referred to as the “Factorial Approach”. In both approaches, the distribution of each independent variable was assessed using a Shapiro-Wilks test for normality and, if found to be significantly non-normal (p ≤ 0.05), was transformed as appropriate to approximate symmetry. In all cases this involved a log_10_ scale transformation, however a square-root transformation was also assessed during preliminary analysis [2,22]. The transformation of independent variables in seasonally driven systems allows for variables to be assessed as a stationary effect, often improving forecasting accuracy. Lags were introduced to each independent variable based on a cross-correlation analysis of the independent variable associated with the dependent variable. These lags help represent the time it takes for RRV to circulate through the mosquito and host populations, and the incubation periods before the onset of symptomatic RRV in humans and its subsequent disease notification. These time lags allow for predictions of RRV notifications to be made for the future. After the introduction of lag periods, pairwise correlations between independent variables were assessed in the independent approach, using Spearman’s correlation coefficient, similar to that of other RRV prediction modelling [1,2,17]. If two independent variables were found to be highly correlated with one another (cut-off of 0.75), the variable with the largest mean absolute correlation with the other independent variables was removed.

Data were split into a training and testing data sets. The training data set included data from July 2000 to June 2012 for Victorian LGAs and from July 1991 to June 2012 for Western Australia. Data from July 2012 to June 2018 for Victoria and Western Australia were then used as the testing data set to validate the models. Five modelling designs were used to predict RRV notifications and outbreaks: these included negative binomial regression, generalised boosted regression, hurdle, generalise additive, and autoregressive integrated moving average (ARIMA) models. Seasonal ARIMA models were initially used; however, the preliminary analysis found the seasonal components of the model did not significantly improve the model predictions. Except for generalised boosted regression models, all models used here represent those which have commonly been used in predicting the transmission and outbreaks of vector-borne diseases, including RRV [23]. For negative binomial regression, generalised boosted regression, hurdle, and generalised additive models, RRV notification data was used as the dependent variable expressed as counts of RRV notifications and human population data of each LGA was then used as an offset term to account for differences in population densities. In the ARIMA models, human notification data were divided by the population at risk and used as the dependent variable.

The Independent Approach used meteorological factors as independent variables in the model. For negative binomial regression, hurdle, generalised additive, and ARIMA models forward and backwards Akaike Information Criterion (AIC) automated stepwise variable selection was used to select the best model fit with the lowest AIC value to make predictions. For the generalised boosted regression models, variables underwent parameter tuning using the relative variable importance, whereby variables with importance equal to zero were excluded from the final model [24,25]. Variable importance was calculated based on the number of times a variable is selected for splitting within the classification decision tree, using weighted squared cumulative reduction in error which is averaged over all regression trees [26,27]. Variable importance is then divided by the highest variable importance to give values between zero and one, with higher values indicating greater importance in the model.

The Factorial Approach uses exploratory factor analysis to find groups of independent variables, “factors”, to be used as independent variables within the final model. To identify factors, a correlation matrix was made of the meteorological variables, allowing for up to nine possible factors. The eigenvalues of this matrix correspond to factors, and those factors with an eigenvalue > 1 were retained for use in the exploratory factor analysis applying an oblique rotation, allowing for correlations between factors, and ordinary least squares to obtain factor scores [28]. Eigenvectors were used as factors and as independent variables with each model. In the generalised additive models for both modelling approaches, a seasonal natural cubic spline was included as a predictor, with knots placed at yearly intervals (every 12 months) to allow for complex seasonality associations in transmission.

The predictive performance of each approach and model type was judged by assessing how well the model was able to predict RRV notifications per 100,000 population, and if the model was able to ‘predict’ an observed RRV outbreak. For the models which predicted counts of RRV notifications, predictions were converted into RRV notifications per 100,000 population. An RRV outbreak was classified using a fixed RRV notification threshold, whereby monthly RRV notifications per 100,000 above the mean plus one standard deviation of the observed RRV notifications per 100,000 for the entire time period for each LGA was classified as an outbreak [2,17]. We initially evaluated three different outbreak thresholds: monthly mean, monthly mean plus one standard deviation, and monthly mean plus two standard deviations to account for variability in RRV notifications. We found that using the monthly mean in many of the Victorian LGAs classified months with a single case as outbreaks and using the monthly mean plus two standard deviations excluded clear distinct outbreak periods and was instead representative of an epidemic threshold (S1 and S2 Figs). For the assessment of how well the model predicted RRV notifications, we evaluated an adjusted R^2^ from a linear regression model of predicted RRV notifications as an independent variable predicting the observed RRV notifications as the dependent variable in the testing portion of the data with a statistical significance having a p-value < 0.05. Predictive model performance for how well predictions matched observed outbreaks were evaluated using sensitivity, specificity, and Matthews correlation coefficient (MCC) [29]. For models predicting outbreaks, where MCC values were equal, the same model type which had the greater adjusted R^2^ was used as the best fit model.

A Jackknife approach was used to assess how sensitive the best fit models were to the training data to obtain 95 percent confidence intervals for each of the predictive performance measures. We randomly resampled 90 percent of our training data 1000 times, creating pseudo-random training data before refitting each best fit model and making predictions on the testing data. Undertaking the Jackknife approach allowed us to obtain the distribution of each model’s respective predictive performance on the testing data and assess how reliant the best fit model’s predictive accuracy and model performance is on the selection the training data sample.

Statistical analysis was undertaken in R (Version 3.5.3, www.r.project.org), using the latest compatible versions of packages ‘mltools, ‘psych’, ‘gam’, ‘caret’, ‘mlbench’, ‘mgcv’, ‘MuMIn’, ‘pscl’, ‘forecast’, ‘gbm’, ‘splines’, ‘MASS’, ‘broom’, ‘zoo’, and ‘car’.

## Results

For the study period, there were a total of 5,307 RRV notifications across all 18 LGAs (Table 1). The range in the number of RRV cases generally reflects the population differences among the sites, differing time lengths in the data available, and frequency of disease outbreaks.

**Fig 2:**
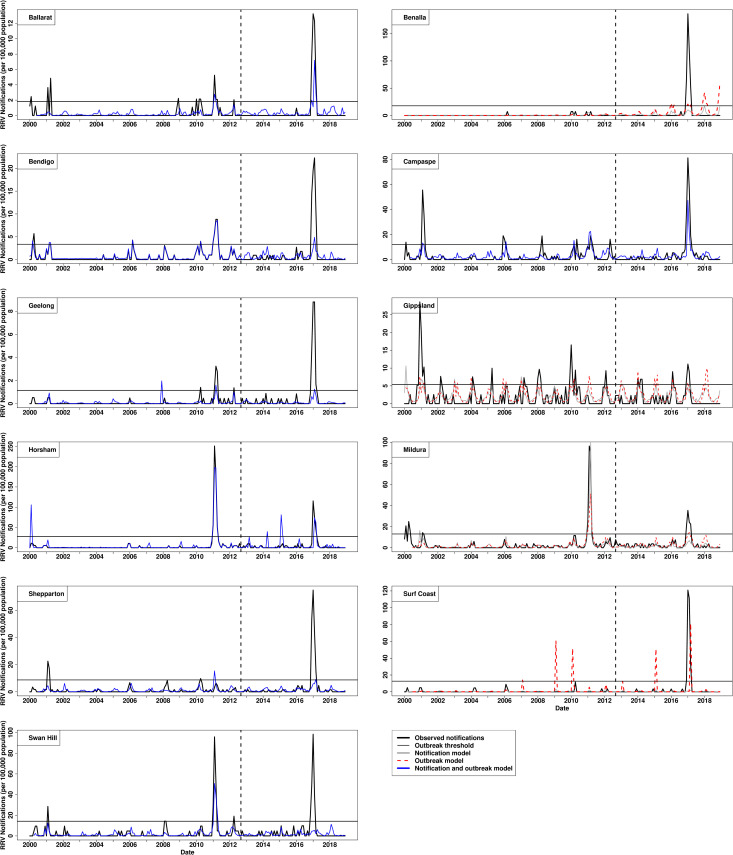
Best fit predictive models of Ross River virus notifications (per 100,000 population) per month for 11 local government areas in Victoria, Australia. Legend: solid black line: observed RRV notifications, solid grey line: model predicted RRV notifications, dotted red line: model predicted notifications used to predict RRV outbreaks, solid light blue line: model predicted RRV notifications used to predict observed notifications and outbreaks, horizontal solid black line: notifications threshold to classify outbreaks, dashed vertical black line: splitting training (left side of line) and testing (right side of line) data.

**Fig 3:**
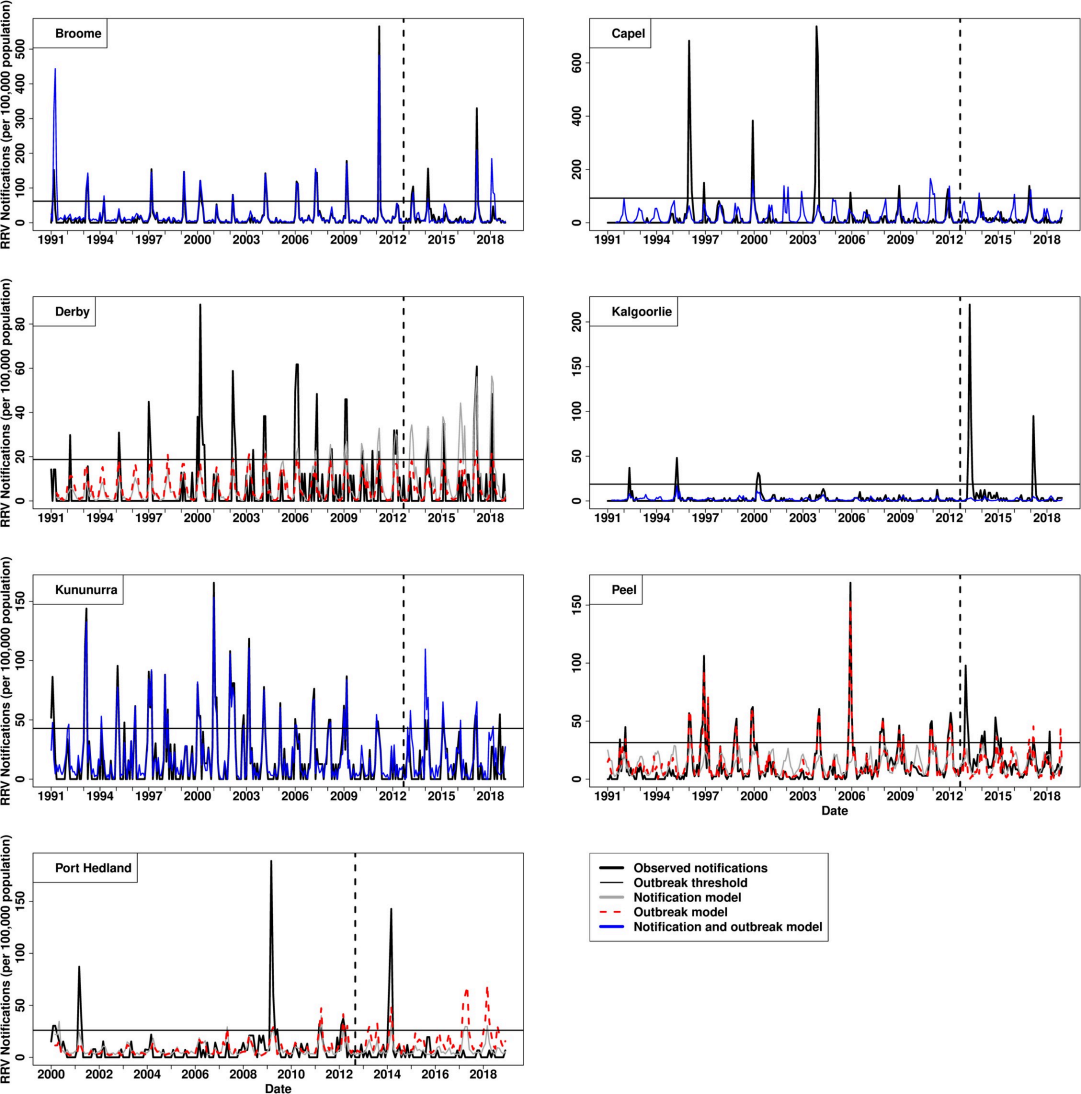
Best fit predictive models of Ross River virus notifications (per 100,000 population) per month for six local government areas in Western Australia. Legend: solid black line: observed RRV notifications, solid grey line: model predicted RRV notifications, dotted red line: model predicted notifications used to predict RRV outbreaks, solid light blue line: model predicted RRV notifications used to predict observed RRV notifications and outbreaks, horizontal solid black line: RRV notifications threshold to classify outbreaks, dash vertical black line: splitting training (left side of line) and testing (right side of line) data.

Of the 18 LGAs, 12 identified the same model type as performing best for predicting outbreaks and RRV notifications, while the remaining six LGAs had two differing model types to separately predict outbreaks and RRV notifications (Tables 1 and 2). Between the two modelling approaches, Independent Approach was found to be the best method for predicting RRV outbreaks and RRV notifications, with more LGAs having a best fit model with this method than that of the Factorial Approach (Table 1). One LGA had a best fit model using the Factorial Approach for predicting both outbreaks and RRV notifications, one LGA used the Factorial Approach for predicting outbreaks while using the Independent Approach for predicting RRV notifications, and one LGA used the Factorial Approach for predicting RRV notifications while using the Independent Approach for predicting RRV outbreaks (Table 1). The predictive models appeared to generally capture the activity in RRV transmission across LGAs (Figs 2 and 3). The mean sensitivity and specificity for a model to correctly identify outbreaks among the LGAs examined were 0.40 and 0.97, respectively (Table 1). The sensitivity and specificity values seen in the models is further supported by having a weak to moderate mean Matthews correlation coefficient (MCC = 0.43) (Table 1). The model’s predictive performance is apparent when predictions are visually plotted against the observed RRV notifications (Figs 2 and 3, for variables included in each best fit model see S1 Table). Ballarat and Campaspe were found to have the best performing model to predict RRV outbreaks with a moderate to strong Matthews correlation coefficient of 0.86 and 0.70 respectively (Table 1). Campaspe in Victoria had the best performing model for predicting RRV notifications when assessing the R-squared coefficient (Table 1). While the models for Swan Hill, Capel, and Kalgoorlie were found to be the poorest at predicting outbreaks. Generalised additive models were found to be the most common best fit predictive model among LGAs for predicting both outbreaks (6/18) and RRV notifications (7/18) (Tables 1 and 2). The best-fit model for predicting outbreaks, after generalised additive models, were generalised boosted regression models (5/18), hurdle models (4/18), and negative binomial regression models (3/18). The best-fit model for predicting RRV notifications, after generalised additive models, were hurdle models (5/18), generalised boosted regression models (4/18), and negative binomial regression models (2/18). ARIMA models were not chosen as a best-fit model for predicting RRV notifications or outbreaks in any LGA. The most identified best fit predictive model among the Victorian LGAs were generalised additive models which were used in seven of the 11 LGAs, while the most identified best-fit model in Western Australia were negative binomial regression and boosted regression models, being used in three of the seven LGAs each. Interestingly, boosted regression models fitted RRV notifications better than the other statistical methods in the training data, but were not the best at predicting RRV notifications or outbreaks (Table 1, Figs 1 and 2).

**Table 2:** Independent and Factorial Approach results by State; Victoria (VIC), and Western Australia (WA) and local government area (LGA) for predicting RRV notifications per 100,000 population (R^2^) and outbreaks (Sn, Sp and MCC). Adjusted R-squared coefficient (R^2^), sensitivity (Sn), specificity (Sn), and Matthews correlation coefficient (MCC) of model performance and predictions. Shading represents the best fit statistical model for predicting notifications (grey); predicting outbreaks (red): and predicting both outbreaks and notifications (blue).

State	LGA	Boosted Regression				Generalised Additive Model				Hurdle Model				Negative Binomial				ARIMA			
		R^2^	Sn	Sp	MCC	R^2^	Sn	Sp	MCC	R^2^	Sn	Sp	MCC	R^2^	Sn	Sp	MCC	R^2^	Sn	Sp	MCC
	***Independent Approach***																				
VIC	Ballarat	0.18	0.50	1.00	0.70	0.53	0.75	1.00	0.86	0.37	0.00	1.00	0.00	0.52	0.25	1.00	0.49	0.07	0.00	1.00	0.00
VIC	Benalla	0.00	0.00	1.00	0.00	0.14	0.00	1.00	0.00	0.12	0.00	1.00	0.00	0.00	0.00	1.00	0.00	0.10	0.00	1.00	0.00
VIC	Bendigo	0.43	0.25	1.00	0.49	0.17	0.75	0.88	0.38	0.27	0.00	1.00	0.00	0.18	0.00	1.00	0.00	0.04	0.00	1.00	0.00
VIC	Campaspe	0.26	0.50	0.97	0.47	0.74	0.50	1.00	0.70	0.76	0.50	1.00	0.70	0.74	0.50	1.00	0.70	0.29	0.25	1.00	0.49
VIC	Geelong	0.18	0.25	1.00	0.49	0.29	1.00	0.84	0.46	0.58	0.25	1.00	0.49	0.26	0.00	1.00	0.00	0.09	0.00	1.00	0.00
VIC	Gippsland	0.02	0.43	0.87	0.24	0.14	0.00	0.99	-0.04	0.13	0.43	0.92	0.31	0.14	0.29	0.93	0.22	0.12	0.00	1.00	0.00
VIC	Horsham	0.14	0.67	0.96	0.49	0.06	1.00	0.83	0.39	0.00	0.00	1.00	0.00	0.06	0.00	1.00	0.00	0.08	0.00	1.00	0.00
VIC	Mildura	0.13	0.50	0.96	0.41	0.40	0.25	1.00	0.49	0.46	0.00	1.00	0.00	0.36	0.00	1.00	0.00	0.27	0.00	1.00	0.00
VIC	Shepparton	0.12	0.00	1.00	0.00	0.30	0.25	1.00	0.49	0.16	0.00	1.00	0.00	0.29	0.00	1.00	0.00	0.11	0.00	1.00	0.00
VIC	Surf Coast	-0.01	0.00	1.00	0.00	0.08	0.00	1.00	0.00	0.01	0.25	0.99	0.33	0.06	0.00	1.00	0.00	0.07	0.00	1.00	0.00
VIC	Swan Hill	-0.01	0.00	0.97	-0.04	0.07	0.00	1.00	0.00	0.05	0.00	1.00	0.00	0.06	0.00	1.00	0.00	0.02	0.00	1.00	0.00
WA	Broome	0.52	0.75	0.95	0.54	0.33	1.00	0.88	0.52	0.42	0.75	0.95	0.54	0.36	0.00	0.97	-0.04	0.19	0.00	1.00	0.00
WA	Capel	0.11	0.00	0.97	-0.02	0.22	0.00	1.00	0.00	0.18	0.00	0.95	-0.03	0.23	0.00	0.96	0.00	0.11	0.00	1.00	0.00
WA	Derby	0.16	0.29	0.93	0.22	0.36	1.00	0.79	0.50	0.28	0.29	0.99	0.40	0.32	0.43	0.99	0.54	0.08	0.00	1.00	0.00
WA	Kalgoorlie	0.03	0.00	1.00	0.00	0.12	0.00	1.00	0.00	0.14	0.00	1.00	0.00	0.15	0.00	1.00	0.00	0.13	0.00	1.00	0.00
WA	Kununurra	0.38	0.80	0.90	0.50	0.35	0.40	0.89	0.21	0.36	0.40	0.90	0.23	0.36	0.40	0.90	0.23	0.07	0.00	1.00	0.00
WA	Peel	0.09	0.00	0.96	-0.08	0.12	0.45	0.78	0.18	0.17	0.00	0.96	-0.08	0.17	0.18	0.94	0.16	0.13	0.00	1.00	0.00
WA	Port Hedland	0.07	0.50	0.95	0.29	0.07	1.00	0.88	0.40	0.18	0.50	0.96	0.33	0.14	0.00	1.00	0.00	0.04	0.00	1.00	0.00
	***Factorial Approach***																				
VIC	Ballarat	-0.01	0.00	0.97	-0.04	0.03	0.00	1.00	0.00	0.02	0.00	1.00	0.00	0.02	0.00	1.00	0.00	0.00	0.00	1.00	0.00
VIC	Benalla	0.00	0.00	1.00	0.00	0.04	0.50	0.89	0.26	0.02	0.00	1.00	0.00	0.02	0.00	1.00	0.00	0.04	0.00	1.00	0.00
VIC	Bendigo	0.00	0.00	1.00	0.00	0.07	0.00	0.97	-0.04	0.02	0.00	1.00	0.00	0.03	0.00	1.00	0.00	0.05	0.00	1.00	0.00
VIC	Campaspe	-0.01	0.00	0.99	-0.03	0.09	0.00	1.00	0.00	0.07	0.00	1.00	0.00	0.08	0.00	1.00	0.00	0.03	0.00	1.00	0.00
VIC	Geelong	-0.01	0.00	0.99	-0.03	-0.01	0.25	0.81	0.03	-0.01	0.25	0.93	0.15	-0.01	0.25	0.97	0.26	0.00	0.00	1.00	0.00
VIC	Gippsland	0.00	0.29	0.82	0.07	0.22	0.14	0.96	0.13	0.21	0.29	0.92	0.19	0.21	0.14	0.93	0.08	0.12	0.00	1.00	0.00
VIC	Horsham	-0.01	0.00	0.93	-0.05	-0.01	1.00	0.63	0.25	-0.01	0.00	0.96	-0.04	-0.01	0.00	1.00	0.00	0.05	0.00	1.00	0.00
VIC	Mildura	-0.01	0.00	0.95	-0.05	-0.01	0.00	0.96	-0.05	-0.01	0.00	0.97	-0.04	-0.01	0.00	0.97	-0.04	0.04	0.00	1.00	0.00
VIC	Shepparton	0.00	0.00	1.00	0.00	0.04	0.00	1.00	0.00	0.04	0.00	1.00	0.00	0.03	0.00	1.00	0.00	0.04	0.00	1.00	0.00
VIC	Surf Coast	-0.01	0.00	1.00	0.00	-0.01	0.00	1.00	0.00	0.02	0.00	1.00	0.00	-0.01	0.00	1.00	0.00	0.02	0.00	1.00	0.00
VIC	Swan Hill	0.00	0.00	0.97	-0.04	-0.01	0.00	0.93	-0.06	-0.01	0.00	0.97	-0.04	-0.01	0.00	0.99	-0.03	0.02	0.00	1.00	0.00
WA	Broome	0.04	0.00	0.97	-0.04	0.03	0.00	0.95	-0.05	0.04	0.00	1.00	0.00	0.03	0.00	1.00	0.00	0.04	0.00	1.00	0.00
WA	Capel	0.13	0.00	0.99	-0.01	0.1	0.00	1.00	0.00	0.14	0.00	1.00	0.00	0.07	0.00	1.00	0.00	0.10	0.00	1.00	0.00
WA	Derby	0.17	0.57	0.96	0.53	0.06	0.86	0.82	0.45	0.05	0.14	0.96	0.13	0.06	0.14	0.97	0.17	0.14	0.00	1.00	0.00
WA	Kalgoorlie	0.01	0.00	1.00	0.00	0.08	0.00	1.00	0.00	0.12	0.00	1.00	0.00	0.10	0.00	1.00	0.00	0.04	0.00	1.00	0.00
WA	Kununurra	0.20	0.20	0.95	0.15	0.21	0.00	0.99	-0.03	0.21	0.20	0.93	0.12	0.21	0.20	0.92	0.10	0.19	0.00	1.00	0.00
WA	Peel	0.09	0.18	0.97	0.24	0.21	0.45	0.82	0.23	0.25	0.00	1.00	0.00	0.24	0.00	0.99	-0.05	0.15	0.00	1.00	0.00
WA	Port Hedland	-0.01	0.50	0.91	0.21	-0.01	0.00	0.93	-0.04	0.00	0.00	1.00	0.00	-0.01	0.00	1.00	0.00	0.02	0.00	1.00	0.00

The predictive performance measures for outbreaks (i.e., sensitivity, specificity, & MCC) were commonly above of the Jackknife 95% confidence interval distribution suggesting the best fit models have greater predictive accuracy when using larger timeseries (Table 1). Interestingly, there were six sites where the best fit model predictions had an adjusted R^2^ outside of the 95% confidence intervals of the Jackknife R^2^ distribution, which included hurdle models and two generalised boosted regression models (Table 1). Similarly, several of the best fit model predictions had an MCC outside of the 95% confidence intervals of the Jackknife MCC distribution (Table 1). The mean difference between the upper and lower 95% confidence intervals across all sites from the Jackknife distribution for R^2^ and the MCC were 0.07 and 0.02 respectively and ranged from 0.015–0.188 for the R^2^ and 0–0.04 for the MCC.

**Fig 4:**
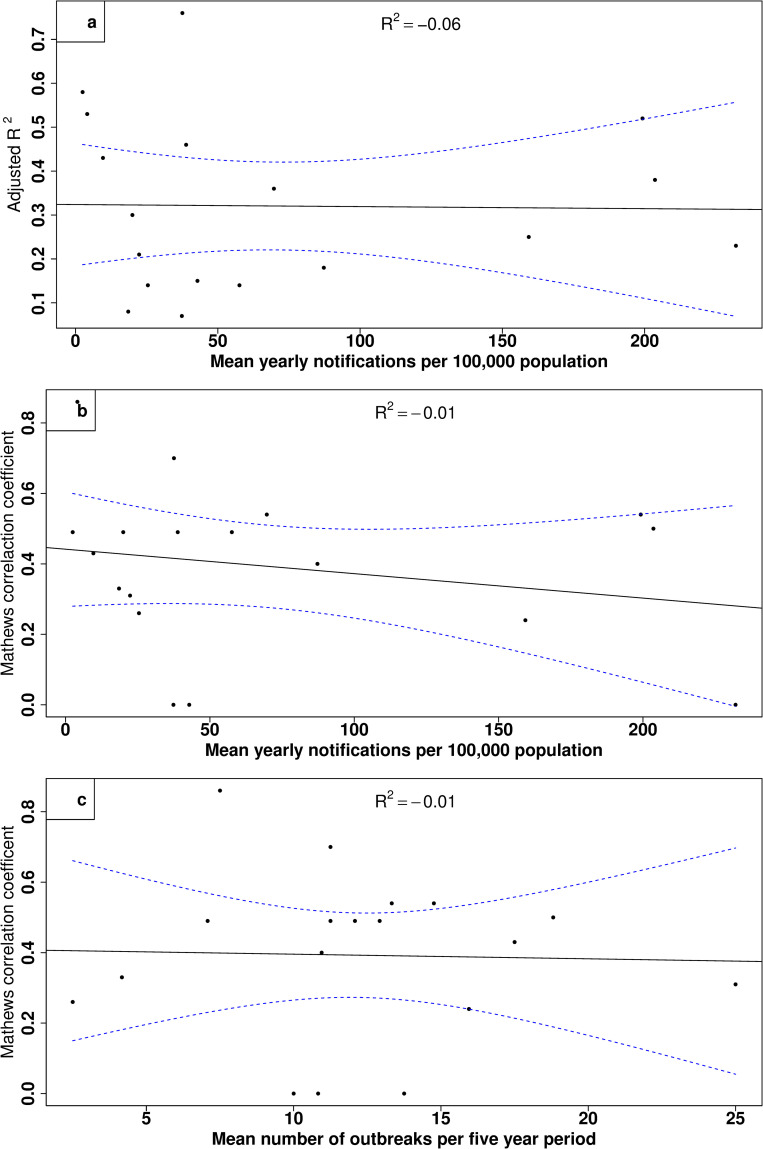
Association between mean annual RRV notifications (per 100,000 population) by LGA with (a) the adjusted R^2^ from a linear regression of the association between predicted RRV notifications and observed RRV notifications in the testing portion of the data; (b) the Matthews Correlation Coefficient from predictions made in the testing portion of the data; and (c) the association between the mean number of months which had a RRV outbreak per five years with the Matthews Correlation Coefficient from predictions. Solid black line is the adjusted R^2^ of the association, and the dashed blue lines show the 95% confidence intervals of the association.

Model performance to predict RRV notifications did not improve with greater annual mean RRV notifications (p-value = 0.94), i.e., greater disease activity (Fig 4A). A model’s ability to predict outbreaks had no association with an LGAs annual mean RRV notifications (p-value = 0.34, Fig 4B) and no significant trend was found in the association between the mean number of outbreaks per five-year period and model performance to predict RRV outbreaks (p-value = 0.35, Fig 4C). Moreover, we found no significant association between greater annual mean RRV notifications with narrower distribution of the predictive performance of MCC (p-value = 0.85) or R^2^ (p-value = 0.95) and no significant association between the mean number of outbreaks per five-year period with a narrower distribution of the predictive performance of MCC (p-value = 0.39) and R^2^ (p-value = 0.91) from the Jackknife pseudo-resampling. An example of this can be seen in Ballarat; despite having the lowest number of RRV notifications among the LGAs examined here, Ballarat had the best predictive model for predicting outbreaks with the highest MCC coefficient (Table 1).

Preliminary analysis investigated three different types of outbreak thresholds where outbreaks were classified if notifications per 100,000 were above the monthly mean, monthly mean plus one standard deviation, and monthly mean plus two standard deviations (S1 and S2 Figs). The threshold of the mean plus one standard deviation was used here. However, preliminary analysis using different outbreak thresholds, such as the monthly mean, for several LGAs led to improved outbreak predictions and different selection of the best fit model for outbreaks (S2 Table). This is illustrated in several of the WA LGAs where outbreak model selected differed and the predictive accuracy was greater using the monthly mean number of RRV notification per 100,000 population as the outbreak threshold versus the monthly mean plus one standard deviation (Tables 1 and S2). Moreover, the confidence intervals of the predictive performance measures for predictive outbreaks from the Jackknife distribution were seen to more commonly be centred around the best fit model estimate in using the outbreak threshold of a monthly mean (Tables 1 and S2).

## Discussion

The transmission of mosquito-borne diseases is complex, with meteorological drivers of disease dynamics varying among geographic and climatic regions. Predictive modelling of the transmission of Ross River virus (RRV) has used multiple statistical approaches for developing forecasting tools [e.g.,6,10,15]. However, the selection of a statistical model over others is rarely discussed or explored, and relative predictive performance comparing models has yet to be assessed in relation to the forecasting of mosquito-borne disease activity. Our study demonstrates the importance of evaluating the selection process (here, Independent vs Factorial) of a statistical model for predicting mosquito-borne diseases, and that the choice of a predictive model can affect the accuracy of disease predictions. To the best of our knowledge, the current study is the first to compare multiple modelling methods for predicting RRV outbreaks and notifications using out-of-sample RRV notifications across multiple Local Government Areas (LGAs) in Australia.

Among the predictive models examined here, there were three statistical model types commonly found to be the best fit model for predicting RRV outbreaks and notifications. Interestingly, out of the 18 LGAs examined, the same type of statistical model for predicting outbreaks and notifications was the best fit for twelve of those LGAs. This demonstrates that predictive models which are used for forecasting RRV notifications may not always be the most ideal for identifying RRV outbreaks or *vice versa*. The best predictive models for predicting outbreaks were found to be generalised additive models and generalised boosted regression models, while, in contrast, the best predictive models for forecasting RRV notifications were generalised additive models and hurdle models. ARIMA models were not found to be a best fit model in any LGA for predicting RRV outbreaks or notifications. This may be in part due to the ARIMA models being inherently sensitive to data containing outliers, in this instance, LGAs which have only a small number of outbreaks present in the data (such as in LGAs where outbreaks were only seen in Victoria during 2011/12 and 2016/17). A seasonal ARIMA model was initially examined during the preliminary analysis, as many of the northern Western Australian LGAs have annual seasonally driven RRV activity. However, the seasonal component consistently led to poorer model predictions and was subsequently dropped. This may be owing to several of the LGAs examined having infrequent and less annual seasonally driven RRV transmission compared with semi-arid and tropical regions in which these models have previously been used, and the seasonal dynamics being partially represented in the meteorological variables [30–32]. A Jackknife approach was used to validate the accuracy of best fit model for each LGA. The predictive performance from the Jackknife approach showed that predictions made by the best fit models for predicting RRV notifications were accurate and the distribution of the predictive performance measures (i.e., adjusted R^2^) to be narrow suggesting the best fit models are reliable estimates when predicting the true risk of disease transmission. Predictive performance of the best fit models for predicting outbreaks was generally better than that of the predictive performance distribution of the Jackknife (i.e., Matthews correlation coefficient), suggesting that the ability to predict outbreaks is improved with a longer timeseries. However, the difference between the predictive performance for outbreaks could also have arisen due to several of the Victorian LGAs having fewer outbreaks in the training data than LGAs with greater RRV activity and with the data partitioning used in the Jackknife causing a distribution lower than that seen in the best fit model trained on the entire time series. There were six LGAs where the best fit model had a greater predictive performance for predicting RRV notifications than when compared with the distribution from the Jackknife. Similarly, there were eleven LGAs where the best fit model had a greater predictive performance for predicting RRV outbreaks than when compared with the distribution from the Jackknife. These LGAs having a greater predictive performance may indicate that for some regions, having a longer/larger timeseries to train a model on leads to greater predictive accuracy. These results suggest that using a k-fold cross-validation method may instead be a more reliable approach in providing greater predictive accuracy by being able to train and test predictive models across the entire data [1].

An RRV outbreak here is defined as a month with a higher number of RRV cases than the monthly mean plus one standard deviation per 100,000, with this outbreak definition commonly being used in RRV predictive modelling [2,17,33]. During preliminary analysis, three outbreak thresholds were explored: notifications above the monthly mean, monthly mean plus one standard deviation, and the monthly mean plus two standard deviations per 100,000 population. From the preliminary analysis using different outbreak thresholds, we demonstrate that the choice of an outbreak threshold can impact upon a best fit model selection and the predictive performance. Using a single outbreak definition across multiple LGAs and geographic regions may overlook many subtle and local differences which can contribute to an outbreak definition. Using a broad outbreak definition where the threshold is set too high could lead to misclassification of an outbreak and a definition not suited to the local RRV transmission ecology, with the models developed potentially being representative of predicting epidemics where the frequency of disease is significantly in excess of what is otherwise expected [34]. There have been multiple outbreak definitions used in modelling RRV [1,2,12,16,17], with further research being needed to advance region-specific outbreak threshold definitions and methods used in RRV predictive modelling to be able to accurately compare predictive performance between studies.

A significant strength to our study is the extensive number of LGAs investigated across multiple climatic regions, and several statistical models evaluated using out-of-sample predictions. This study, to the best of our knowledge, is the first to examine and evaluate the predictive performance of multiple predictive statistical modelling techniques for forecasting RRV activity. Common methods used to evaluate RRV outbreaks have relied upon accuracy, sensitivity, and specificity measures which have limitations of a model’s ability to predict a disease outbreak [1,3,6,12,16,18,29]. In addition to sensitivity and specificity, we used a Matthews correlation coefficient (MCC), which is more robust, as it is calculated based on true positives, false negatives, true negatives, and false positives [29]. The advantage of using MCC to evaluate predictions is that a high quality MCC score is only generated if predictions are correctly classified, in this instance, correctly predicting when there is and is not an RRV outbreak. This allows for a robust and certain means to assess model predictions of binary outcomes, such as RRV outbreaks, where there is imbalance between predictive categories. For example, the LGA of Campaspe in Victoria had a moderate to strong MCC in the best fit model to predict observed RRV outbreaks, however it had a relatively poor sensitivity coefficient, but had high specificity and did not predict outbreaks when there were none. In contrast, the best fit model in the LGA of Port Hedland had relatively high sensitivity and specificity but only had a weak to moderate MCC coefficient as it often predicted outbreaks when there were none. Using sensitivity and specificity alone, Port Hedland would have ranked as one of the best fit models examine here, however using MCC as our predictive measure, the over prediction of outbreaks is taken into consideration and a more robust assessment can be made. The method used here could then be used as a framework when developing more robust mosquito-borne disease predictive models that also use meteorological independent variables for deterministic and predictive disease modelling.

The accuracy of predictive modelling of RRV, as well as of other mosquito-borne diseases, has often been thought to be better in areas with greater disease notifications. However, surprisingly, among the LGAs investigated here, we found no association with a model’s ability to predict RRV notifications in LGAs with more frequent RRV outbreaks or with greater RRV notifications, and no association in accurately predicting RRV outbreaks in LGAs which have a greater yearly mean number of notifications of RRV. Predictive modelling of RRV in the past has shown models forecasting out-of-sample RRV transmission to be less accurate in areas with fewer RRV notifications and outbreaks [1,2,18]. Instead, here we found the best performing model which scored the highest in predicting outbreaks was in an LGA which had the lowest number RRV notifications. Our results suggest that poor predictive performance of RRV notifications may instead be in part due to the use of inappropriate model selection methods.

Supplementing RRV predictive models with mosquito surveillance data in most instances improves notification and outbreak predictions [3,16–18]. However, mosquito surveillance is time and labour intensive, often being too expensive for many LGAs to undertake, particularly in regional areas of Australia. Readily available meteorological information, on the other hand, offers an inexpensive means to model and thereby predict disease transmission and inform public health organisations of future disease events.

Owing to differences in geographic host and vector life-history traits, transmission dynamics in response to meteorological drivers differ between climatic regions [1,12]. We found LGAs in semi-arid and temperate climates had different best fit statistical model types, generalised boosted regression models and generalised additive models respectively. Generalised additive models and negative binomial regression models were the second most used statistical model type in semi-arid and temperate climates, respectively. Epidemiological predictive models have utilised variable selection methods to determine site-specific factors for forecasting RRV transmission, which can then inform public health decision-making. Our findings suggest that in areas where mosquito surveillance is unavailable, statistical model selection may be able to provide improved disease predictive surveillance for public health management.

Meteorological factors often have temporal correlations with one another, for instance, maximum and minimum temperatures generally follow similar temporal trends. The correlation between meteorological factors can cause multicollinearity in statistical models, potentially biasing the effect an independent variable has on explaining or predicting disease. In predicting RRV, the common occurrence of multicollinearity between meteorological independent variables has frequently led to the omission of variables in deterministic and predictive models [1,2,12,35–38]. However, by excluding explanatory independent variables, information specific to the occurrence to seasonal or sporadic outbreaks may be overlooked. Factor Analysis using principle component analysis allows for the inclusion of all related meteorological factors without having multicollinearity, and this is achieved through using eigenvectors as independent variables, from factor scores which have eigenvalues greater than one [28,39].

Interestingly, there were only three LGAs in which the Factorial Approach fitted better than the Independent Approach, with one LGA fitting a model for predicting outbreaks, one for predicant RRV notifications, and one for predicting both RRV notifications and outbreaks. We speculate that the use of factor scores may reduce the susceptibility of the biological dynamics and responses to specific meteorological conditions on disease transmission. For instance, RRV has specific thermal limits, which promote or inhibit viral transmission [40]. Moreover, rainfall has on numerous occasions been shown to be a positive predictor of RRV notifications, with monthly rainfalls exceeding a threshold increasing the likelihood of an outbreak or disease incidence [1,2,6,15–18,30]. The muddling of these specific responses through a factorial representation may overshadow the subtle nuance of environmental and meteorological events and their effect on RRV transmission.

Among our results, we found that generalised boosted regression models had a better model fit to the training data than that of the other models evaluated. Despite this, generalised boosted regressions did not provide as good predictive accuracy and precision for forecasting RRV notifications and outbreaks when assessing the model on testing data. This may suggest that in describing deterministic pathways of previous RRV transmission, generalised boosted regression may help to explain subtle meteorological drivers leading to outbreaks, while for predictive forecasting, the decision trees made when training a model may restrict the forecast flexibility in a time series setting when ecological change in vector and host populations occur which influence RRV transmission.

This study focused on assessing predictive model performance and has not discussed which independent variables were important within each LGA, or the biological and ecological implications of the statistical models. Future studies could explore and compare independent variables used within each statistical model and the factoring of meteorological variables in the Factorial Approach. Furthermore, comparisons could be made between models that include mosquito surveillance and meteorological data and those only using meteorological data alone, to evaluate how well our approach closes the gap in improving predictive capabilities. Moreover, we do not assess the deterministic characteristics of what meteorological variables were associated with RRV notifications and how this differed between LGAs. Therefore, we do not make any inferences on the meteorological drivers which lead to changes in RRV transmission across the LGAs. Here we used estimated date of RRV symptom onset as our outcome of interest; using a back-calculated date of the likely date of exposure by incorporating an expected intrinsic incubation period may further improve model predictive accuracy and be more representative of when RRV transmission is occurring. Other factors likely influencing the accuracy of modelling RRV transmission and subsequent predictive performance are changes in the rate of under- and over-reporting and false positive testing [41–43]. We make no attempt to estimate or control for these parameters. While these factors influence accurately modelling the true extent of disease infections and transmission within populations, using disease surveillance data we have available allows for reliable temporal trends in disease dynamics to be predicted and used in public health decision making.

There are multiple environmental, meteorological, biological, socioeconomic, geographical, host, and vector components which contribute to the transmission dynamics of RRV [10,36,44]. Factors included in the predictive models developed here use meteorological data which are readily accessible without the need for extensive data requests or data gathering processes, allowing for the approach used here to be easily replicated and integrated into predictive disease surveillance systems. However, a caveat to this approach is the omission of variables that have previously been found to be important in the transmission of RRV among regions studied here. For example, variation in tide heights, river flow and height, and climatic conditions (e.g., Southern Oscillation Index) which are known to be associated with increases in mosquito breeding and potential host movement which can lead to greater RRV transmission [1,2,10,17,18,43]. Moreover, mosquito and host species vary between the LGAs examined here. For instance, mosquito populations along coastal LGAs are likely to include halotolerant species while inland areas typically have freshwater breeding mosquitoes. Mosquitoes species communities in North Western parts of Western Australia can include *Culex annulirostris*, a freshwater breeding mosquito, and *Aedes vigilax*, a saltmarsh breeding mosquito [8]. Inland areas of Victoria include *Cu*. *annulirostris* and *Aedes camptorhynchus*, a saltmarsh breeding mosquito [2,17]. Vector and host dynamics play an integral role in shaping the dynamics in disease transmission systems. In models that do not include mosquito surveillance or host information, the differences in mosquito and host species communities are likely represented during variable selection of climatic and meteorological factors which influence these ecological and biological interactions. Within our variable selection process, variables may have undergone a logarithmic transformation which may lead to models being overfitted. As our aim was to develop and assess forecast models, we are less focused on the climatic epidemiological implications in RRV transmission.

In conclusion, we present new approaches to developing and improving environmental and meteorologically driven mosquito-borne disease early warning forecasting tools. Our findings show that predictive models developed for forecasting disease notifications may not always be suited for forecasting disease outbreaks or *vice versa*. When developing a mosquito-borne disease predictive model for forecasting disease outbreaks and disease notifications, generalised additive models and generalised boosted regression models, and generalised additive models and hurdle models were most often selected as the best fit models, respectively, and are recommended as an initial model when developing future RRV predictive models. However, we demonstrate that in some regions, the model type used needs further discrimination to achieve reliable and accurate predictions. The use and evaluation of predictive performance of statistical models for mosquito-borne diseases have largely been neglected, with research typically only presenting and discussing a single modelling approach. Our findings highlight the importance of the selection of a statistical model used for out-of-sample predictive modelling in RRV. We demonstrate that a model’s ability to predict RRV outbreaks or notifications is not greater in areas with higher yearly RRV notifications. Our approach used in this research aims to provide a new perspective and framework in accurately predicting RRV using only meteorological data where mosquito surveillance information is not available. By using this approach, disease forecast systems can be established to aid in public health decision making and allow for timely and targeted mitigation activities to be carried out effectively to reduce the significant burden of RRV disease in Australia.

## Supporting information

S1 Fig Best fit predictive models of Ross River virus notifications (per 100,000 population) per month for 11 local government areas in Victoria, Australia. Legend: solid black line: observed RRV notifications, solid grey line: model predicted RRV notifications, dotted red line: model predicted notifications used to predict RRV outbreaks, solid light blue line: model predicted RRV notifications used to predict observed notifications and outbreaks, horizontal solid black lines: notifications threshold to classify outbreaks (monthly mean, monthly mean plus one standard deviation, monthly mean plus two standard deviations), dashed vertical black line: splitting training (left side of line) and testing (right side of line) data.
(EPS)

S2 Fig Best fit predictive models of Ross River virus notifications (per 100,000 population) per month for six local government areas in Western Australia. Legend: solid black line: observed RRV notifications, solid grey line: model predicted RRV notifications, dotted red line: model predicted notifications used to predict RRV outbreaks, solid light blue line: model predicted RRV notifications used to predict observed RRV notifications and outbreaks, horizontal solid black lines: RRV notifications threshold to classify outbreaks (monthly mean, monthly mean plus one standard deviation, monthly mean plus two standard deviations), dash vertical black line: splitting training (left side of line) and testing (right side of line) data.
(EPS)

S1 Table Variables used within each best fit model for each Local Government Area (LGA). ARIMA = auto-regressive moving average model; GAM = generalised additive model; BR = generalised boosted regression; NB = negative binomial regression; and Hurdle = hurdle regression. Models with a “*” following the model type used the Factorial Approach. Variables followed by a “$” represents a variable that did not undergo a log10 transformation. Variable acronyms are as follows MSLP = mean sea level pressure; VP = mean vapor pressure; Rhmax/min = maximum and minimum relative humidity; Tmax/min = maximum and minimum temperature; EVA = Morton’s areal actual evapotranspiration; EPP = Morton’s areal potential evapotranspiration; and F1, F2, and F3 are Eigenvectors with variables names within each bracket indicating variables included in the Eigenvector.
(DOCX)

S2 Table Best fit model predictive performance of RRV notifications and outbreaks in local government areas (LGA) in Victoria (VIC), and Western Australia (WA) by LGA climate using the monthly mean number of RRV notifications by 100,000 population as the outbreak threshold. The total number of RRV notifications (Cases), the best model used for predicting RRV notifications, adjusted R-squared coefficient (R^2^), the best model used for predicting outbreaks, sensitivity (Sn), specificity (Sp), and Matthews correlation coefficient (MCC). ARIMA = auto-regressive moving average model; GAM = generalised additive model; BR = generalised boosted regression; NB = negative binomial regression; and Hurdle = hurdle regression. Ninety five percent confidence intervals (95% CI) are given of the distribution of each predictive performance measure from Jackknife pseudo-random sampling using the respective best fit model. Models with a “*” following the model type used the Factorial Approach. See Table 2 for a comparison of how close modelling methods were to one another for predicting RRV notifications and outbreaks.
(DOCX)
