# Supplementary material for: Optimising predictive modelling of Ross River virus using meteorological variables
Source: PLoS Negl Trop Dis. 2021 Mar 9;15(3):e0009252. doi: 10.1371/journal.pntd.0009252 (PMC7978384; doi:10.1371/journal.pntd.0009252)
Supplement: S1 Table — ARIMA = auto-regressive moving average model; GAM = generalised additive model; BR = generalised boosted regression; NB = negative binomial regression; and Hurdle = hurdle regression. Models with a “*” following the model type used the Factorial Approach. Variables followed by a “$” represents a variable that did not undergo a log10 transformation. Variable acronyms are as follows MSLP = mean sea level pressure; VP = mean vapor pressure; Rhmax/min = maximum and minimum relative humidity; Tmax/min = maximum and minimum temperature; EVA = Morton’s areal actual evapotranspiration; EPP = Morton’s areal potential evapotranspiration; and F1, F2, and F3 are Eigenvectors with variables names within each bracket indicating variables included in the Eigenvector. (DOCX) [file pntd.0009252.s003.docx]

**S1 Table:** Variables used within each best fit model for each Local Government Area (LGA). ARIMA = auto-regressive moving average model; GAM = generalised additive model; BR = generalised boosted regression; NB = negative binomial regression; and Hurdle = hurdle regression. Models with a “*” following the model type used the Factorial Approach. Variables followed by a ^“$”^ represents a variable that did not undergo a log_10_ transformation. Variable acronyms are as follows MSLP = mean sea level pressure; VP = mean vapor pressure; Rh^max/min^ = maximum and minimum relative humidity; T^max/min^  = maximum and minimum temperature; EVA = Morton’s areal actual evapotranspiration; EPP = Morton’s areal potential evapotranspiration; and F1, F2, and F3 are Eigenvectors with variables names within each bracket indicating variables included in the Eigenvector.

|  | Notification Models | | Outbreak Models | |
| --- | --- | --- | --- | --- |
| LGA | Model | Variables | Model | Variables |
| Ballarat | GAM | Rainfall, MSLP | GAM | Rainfall, MSLP |
| Benalla | GAM | VP | GAM* | F1 (VP, T^max^, T^min^, Rh^min^, EPA, EPP, MSLP), F2 (Rh^max^, Rainfall) |
| Bendigo | BR | VP, Rainfall, MSLP, Rh^min^ | BR | VP, Rainfall, MSLP, Rh^min^ |
| Campaspe | Hurdle | Rainfall, VP | Hurdle | Rainfall, VP |
| Geelong | Hurdle | Rainfall, T^min^, Rh^max^ | Hurdle | Rainfall, T^min^, Rh^max^ |
| Gippsland | GAM* | F1 (T^max^, T^min^, VP, EPA, EPP) F2 (Rainfall, Rh^max$^, Rh^min^), F3 (MSLP^$^) | Hurdle | VP, Rh^min^ |
| Horsham | BR | VP, Rainfall, T^min^, MSLP | BR | VP, Rainfall, T^min^, MSLP |
| Mildura | Hurdle | Rainfall, VP, Rh^max^ | GAM | Rainfall, VP, Rh^max^ |
| Shepparton | GAM | Rainfall, VP, Rh^min^, MSLP | GAM | Rainfall, VP, Rh^min^, MSLP |
| Surf Coast | GAM | VP, MSLP | Hurdle | VP, MSLP, EPP |
| Swan Hill | GAM | Rainfall, VP | GAM | Rainfall, VP |
| Broome | BR | Rh^max^, Rh^min^, EPP, Rainfall, T^max^ | BR | Rh^max^, Rh^min^, EPP, Rainfall, T^max^ |
| Capel | NB | Rainfall, T^min^, Rh^min^ | NB | Rainfall, T^min^, Rh^min^ |
| Derby | GAM | Rainfall, T^min^, EPP | NB | Rainfall, T^min^, EPP |
| Kalgoorlie | NB | Rainfall, VP, EPA | NB | Rainfall, VP, EPA |
| Kununurra | BR | EPP, T^min^ | BR | EPP, T^min^ |
| Peel | Hurdle* | F1 (Rainfall, T^max^, T^min^, Rh^max^, Rh^min^, EPA, EPP), F2 (VP) | BR* | F1 (Rainfall, T^max^, T^min^, Rh^max^, Rh^min^, EPA, EPP), F2 (VP) |
| Port Hedland | Hurdle | Rainfall, Rh^min$^ | GAM | Rainfall, Rh^min$^ |
