# Supplementary material for: Optimising predictive modelling of Ross River virus using meteorological variables
Source: PLoS Negl Trop Dis. 2021 Mar 9;15(3):e0009252. doi: 10.1371/journal.pntd.0009252 (PMC7978384; doi:10.1371/journal.pntd.0009252)
Supplement: S2 Table — The total number of RRV notifications (Cases), the best model used for predicting RRV notifications, adjusted R-squared coefficient (R2), the best model used for predicting outbreaks, sensitivity (Sn), specificity (Sp), and Matthews correlation coefficient (MCC). ARIMA = auto-regressive moving average model; GAM = generalised additive model; BR = generalised boosted regression; NB = negative binomial regression; and Hurdle = hurdle regression. Ninety five percent confidence intervals (95% CI) are given of the distribution of each predictive performance measure from Jackknife pseudo-random sampling using the respective best fit model. Models with a “*” following the model type used the Factorial Approach. See Table 2 for a comparison of how close modelling methods were to one another for predicting RRV notifications and outbreaks. (DOCX) [file pntd.0009252.s004.docx]

**S2 Table:** Best fit model predictive performance of RRV notifications and outbreaks in local government areas (LGA) in Victoria (VIC), and Western Australia (WA) by LGA climate using the monthly mean number of RRV notifications by 100,000 population as the outbreak threshold. The total number of RRV notifications (Cases), the best model used for predicting RRV notifications, adjusted R-squared coefficient (R^2^), the best model used for predicting outbreaks, sensitivity (Sn), specificity (Sp), and Matthews correlation coefficient (MCC). ARIMA = auto-regressive moving average model; GAM = generalised additive model; BR = generalised boosted regression; NB = negative binomial regression; and Hurdle = hurdle regression. Ninety five percent confidence intervals (95% CI) are given of the distribution of each predictive performance measure from Jackknife pseudo-random sampling using the respective best fit model. Models with a “*” following the model type used the Factorial Approach. See Table 2 for a comparison of how close modelling methods were to one another for predicting RRV notifications and outbreaks.

|  |  |  | Outbreak Models | | | |
| --- | --- | --- | --- | --- | --- | --- |
| State | LGA | Climate | Best model | Sn (95% CI) | Sp (95% CI) | MCC (95% CI) |
| VIC | Ballarat | Temperate | ARIMA | 0.20 (0.60-0.80) | 1.00 (0.63-0.97) | 0.44 (0.22-0.47) |
| VIC | Benalla | Semi-arid | GAM | 0.83 (0.67-0.83) | 0.76 (0.57-0.88) | 0.35 (0.22-0.39) |
| VIC | Bendigo | Semi-arid | NB* | 0.39 (0.22-0.56) | 0.83 (0.75-0.90) | 0.23 (0.08-0.33) |
| VIC | Campaspe | Semi-arid | NB | 0.54 (0.38-0.54) | 0.85 (0.82-0.88) | 0.35 (0.26-0.37) |
| VIC | Geelong | Semi-arid | Hurdle | 0.31 (0.13-0.38) | 0.94 (0.94-0.98) | 0.31 (0.17-0.46) |
| VIC | Gippsland | Semi-arid | GAM* | 0.65 (0.62-0.81) | 0.73 (0.39-0.73) | 0.37 (0.18-0.38) |
| VIC | Horsham | Semi-arid | BR | 0.35 (0.30-0.52) | 0.89 (0.62-0.87) | 0.28 (0.08-0.22) |
| VIC | Mildura | Temperate | Hurdle | 0.29 (0.25-0.33) | 0.96 (0.93-0.96) | 0.37 (0.29-0.37) |
| VIC | Shepparton | Temperate | GAM | 0.89 (0.66-1.00) | 0.78 (0.67-0.87) | 0.47 (0.34-0.51) |
| VIC | Surf Coast | Temperate | BR | 0.44 (0.00-0.11) | 0.80 (1.00-1.00) | 0.18 (0.00-0.20) |
| VIC | Swan Hill | Temperate | GAM | 0.35 (0.35-0.45) | 0.84 (0.79-0.93) | 0.21 (0.17-0.32) |
| WA | Broome | Semi-arid | NB | 0.76 (0.70-0.82) | 0.89 (0.89-0.89) | 0.61 (0.57-0.66) |
| WA | Capel | Temperate | NB | 0.91 (0.55-0.91) | 0.73 (0.72-0.76) | 0.46 (0.22-0.48) |
| WA | Derby | Semi-arid | Hurdle | 0.67 (0.62-0.71) | 0.74 (0.70-0.75) | 0.37 (0.33-0.39) |
| WA | Kalgoorlie | Temperate | GAM* | 0.39 (0.00-0.28) | 0.92 (0.90-1.00) | 0.36 (-0.11-0.25) |
| WA | Kununurra | Semi-arid | BR | 0.73 (0.68-0.73) | 0.82 (0.79-0.84) | 0.52 (0.46-0.51) |
| WA | Peel | Temperate | NB* | 0.74 (0.68-0.77) | 0.77 (0.72-0.81) | 0.50 (0.46-0.53) |
| WA | Port Hedland | Semi-arid | ARIMA* | 0.78 (0.44-0.89) | 0.43 (0.26-0.45) | 0.14 (-0.13-0.13) |
| Mean |  |  |  |  |  |  |
| Overall |  |  |  | 0.57 | 0.82 | 0.36 |
| VIC |  |  |  | 0.48 | 0.85 | 0.32 |
| WA |  |  |  | 0.71 | 0.75 | 0.42 |
| STDEV |  |  |  |  |  |  |
| Overall |  |  |  | 0.22 | 0.13 | 0.12 |
| VIC |  |  |  | 0.23 | 0.09 | 0.09 |
| WA |  |  |  | 0.22 | 0.07 | 0.12 |
